# Decompression with interbody fusion versus decompression alone for degenerative lumbar diseases: A meta-analysis

**DOI:** 10.1371/journal.pone.0330926

**Published:** 2025-08-26

**Authors:** Yuxian Chen, Shenglin Lei, Wei Lin, Yilin Huang, Pinying Cheng, Shuling Gu, Dongping Wang

**Affiliations:** 1 The First Clinical Medical School, Guangzhou University of Chinese Medicine, Guangzhou, Guangdong, China; 2 Shenzhen Clinical Medical School, Guangzhou University of Chinese Medicine, Guangzhou, Guangdong, China; 3 The Second Clinical Medical School, Guangzhou University of Chinese Medicine, Guangzhou, Guangdong, China; 4 The Fifth Clinical Medical School, Guangzhou University of Chinese Medicine, Guangzhou, Guangdong, China; 5 Acupuncture Moxibustion and Rehabilitation Clinical Medical School, Guangzhou University of Chinese Medicine, Guangzhou, Guangdong, China; Aichi Prefectural Mikawa Aoitori Medical and Rehabilitation Center for Developmental Disabilities, JAPAN

## Abstract

**Objective:**

To appraise the clinical effectiveness and complications of two surgical approaches, namely decompression alone (DA) versus decompression with interbody fusion (DF), in managing degenerative lumbar diseases (DLD).

**Methods:**

As of July 1, 2024, an exhaustive search identified all randomized controlled studies and cohort studies comparing DA and DF in DLD management. Relevant data were extracted using strict criteria, and study quality was assessed via the Newcastle-Ottawa Scale and Cochrane Collaboration’s tool. The extracted outcomes encompassed a range of measures, including operative duration, intraoperative hemorrhage, hospitalization length, time to ambulation, short form 12 physical component score (SF12-PCS), low back pain visual analog scale (VAS) score, leg pain VAS score, Oswestry disability index (ODI), Japanese orthopedic association (JOA) score, EuroQol five dimensions (EQ-5D), incidence of complications, reoperation rate, and Odom’s criteria.

**Results:**

A total of 35 articles were included in this study, involving 12,030 patients. Of these, 7,442 patients were in the DA group, while 4,588 were in the DF group. Operative duration was shorter (MD = −89.09, 95%CI −92.71, −85.47, *P* < 0.00001), intraoperative hemorrhage was less (MD = −242.26, 95%CI −252.16, −232.36, *P* < 0.00001), hospitalization length was shorter (MD = −2.36, 95%CI −2.59, −2.14, *P* < 0.00001), and time to ambulation was reduced (MD = −10.49, 95%CI −12.52, −8.46, *P* < 0.00001) in the DA group than in the DF group. At the final follow-up for ODI, the DF group demonstrated statistically superior outcomes compared to the DA group (MD = 1.28, 95%CI 0.35, 2.21, **P* *= 0.007). Data revealed no significant differences in SF12-PCS, JOA score, back pain VAS score, leg pain VAS score, final follow-up EQ-5D, reoperation rates, complication rates, and Odom’s criteria (**P* *> 0.05).

**Conclusion:**

When treating DLD, DA offers more favorable outcomes in terms of operative duration, intraoperative hemorrhage, hospitalization length, and time to ambulation. These findings suggest that DA should be considered the preferred surgical approach for most DLD patients, unless specific indications for fusion exist. Clinicians should tailor decisions to each surgery’s specifics to optimize patient outcomes.

**Trial registration:**

PROSPERO registration number: CRD42024580975.

## Introduction

Degenerative lumbar diseases (DLD) are prevalent chronic health issues, predominantly affecting the elderly but increasingly observed in younger populations [1]. These conditions are typically marked by lower back and leg pain, along with impaired physical function, resulting in long-term discomfort and a substantial financial burden on patients [2,3].

Degenerative lumbar disc herniation (DLDH), degenerative lumbar spinal stenosis (DLSS), and degenerative lumbar spondylolisthesis (DLS) are the three most common types of DLD. Previous studies have shown DLDH leads to sciatica with an annual prevalence of 1–5%, while the lifetime prevalence can reach up to 43% [4]. Furthermore, according to radiological criteria, 21% of individuals over the age of 60 are diagnosed with DLSS [5]. DLS occurs when one vertebra slips over another, potentially causing central canal stenosis and instability. It affects approximately 4.1% of the general population and is rare before the age of 50 [6]. Conservative treatment is generally recommended for patients without significant neurological deficits, as it often leads to symptom relief. However, surgical intervention may be necessary when pain intensifies, new neurological deficits develop, or if conservative treatment proves ineffective after three months.

Recently, decompression alone (DA) and decompression with interbody fusion (DF) are established surgical interventions extensively employed in clinical settings for treating DLD [7]. Despite their acceptance, comparative studies on the effectiveness of DA versus DF reveal inconsistent outcomes, thereby perpetuating uncertainty regarding the optimal surgical approach for individual patients [8–10]. Consequently, both patients and healthcare providers face challenges in determining the most appropriate option between DA and DF.

This investigation endeavors to consolidate extant research, assessing the comparative effectiveness and safety profiles of DA and DF, with the objective of informing forthcoming surgical decisions in the context of DLD management.

## Methods

### Registration ID

This meta-analysis was conducted in accordance with the PRISMA guidelines and was registered in the PROSPERO database under registration number CRD42024580975 [11]. The PRISMA checklist is available in S1 File.

### Literature search strategy

English databases, including the Cochrane Library, PubMed, Web of Science, and Embase, were systematically searched using keywords such as fusion, decompression, lumbar spinal stenosis, lumbar disc herniation, lumbar spinal canal stenosis, intervertebral disk displacement, spondylolisthesis, and olisthesis. The search spanned from the inception of each database to July 1, 2024. The search strategy is provided in the S2 File.

### Inclusion and exclusion criteria

The inclusion criteria were as follows: 1) patients undergoing DA (partial laminectomy and decompression of compressed structures, such as protruding disc fragments, hypertrophic ligaments, and ossified small joint surfaces) or DF (decompression and insertion of pedicle screws between adjacent vertebrae for fixation, with selective implantation of bone grafts using any approach) for the management of DLD; 2) outcomes included operative duration, intraoperative hemorrhage, hospitalization length, time to ambulation, short form 12 physical component score (SF12-PCS), low back pain visual analog scale (VAS) score, leg pain VAS score, Oswestry disability index (ODI), Japanese orthopedic association (JOA) score, EuroQol five dimensions (EQ-5D), incidence of complications, reoperation rate, and Odom’s criteria; 3) literature types included randomized controlled trials (RCTs), retrospective studies, and prospective studies with at least 6 months of postoperative follow-up.

The exclusion criteria were as follows: 1) patients with combined lumbar spinal infectious diseases, tumor diseases, and bleeding diseases; 2) non-primary lumbar surgeries; 3) reviews, conferences, expert opinions, case reports, animal experiments, and unavailable literature; 4) non-English language publications; 5) studies lacking complete outcome data.

### Statistics extraction and collection

Two researchers independently screened literature and extracted data based on predefined criteria. Collected baseline characteristics included author, year, country, study design, disease type, sample size, mean age, and follow-up duration. Outcome measures matched the inclusion criteria. Data were standardized: operative time (minutes), blood loss (milliliters), hospital stay and ambulation time (days), and VAS score for back and leg pain (0–10, with 10 being most severe). Results included patients rated as excellent or good by Odom’s criteria. Discrepancies encountered during the data extraction process were addressed by an independent senior researcher.

### Quality assessment

Two investigators independently assessed bias risk, followed by cross-validation for consistency. The Newcastle Ottawa Scale (NOS) was employed to appraise cohort studies, encompassing aspects of study design, comparability, and exposure [12]. Each study could attain a maximum score of nine points, categorized into low-quality (0–3 points), moderate-quality (4–6 points), and high-quality (7–9 points) classifications. The Cochrane Collaboration’s tool [13] was utilized in evaluating RCTs. In instances where discrepancies arose during the quality assessment, they were reconciled by a third senior researcher.

### Statistical analysis

A meta-analysis of observational indicators was performed using Review Manager 5.4 software. For continuous variables, such as ODI and JOA, the mean difference (MD) was used as the effect measure. For dichotomous variables, such as complications and reoperation, the odds ratio (OR) was employed as the effect measure. All effect measures were provided with a 95% confidence interval (CI). Heterogeneity analysis among studies was appraised using the *I*^*2*^ statistic. If *I*^*2 *^< 50%, heterogeneity was considered low, and the fixed effect model was chosen. If *I*^*2 *^> 50%, heterogeneity was considered significant, and a random-effects model was selected. Subgroup analysis was conducted to identify potential sources of heterogeneity, and sensitivity analysis was performed to minimize heterogeneity as much as possible. When heterogeneity was high, sensitivity analysis was conducted by excluding studies one by one until it dropped below 50%. If heterogeneity remained high, a random-effects model or subgroup analysis was applied. Bias for each study was assessed using funnel plots.

## Results

### Literature search results and literature screening flow

**Fig 1:**
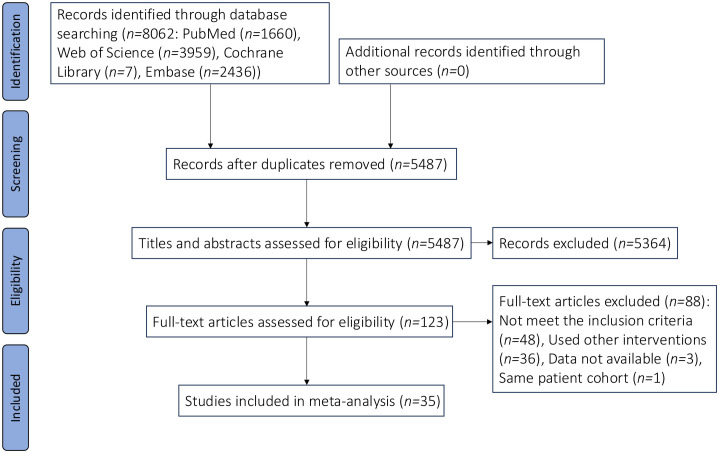
Flow diagram of study selection.

From an initial pool of 8,062 records sourced across four databases using predefined search terms, 2,575 duplicates were identified and removed. Applying our inclusion criteria, we screened titles and abstracts to exclude a further 5,364 records. Subsequent full-text examination of the remaining 123 articles resulted in the selection of 35 eligible studies [8–10,14–45], involving a total of 12,030 patients (Fig 1). This cohort comprised 7,442 patients in the DA group and 4,588 in the DF group. A summary of the fundamental characteristics for these 35 studies is presented in Table 1.

**Table 1:** Characteristics of the included literature.

NO	Author (year)	Country	Study Design	Disease type	DA/DF			
					Females (N)	Gender (N)	Age (years)	Follow-up (months)
1	Aleksandra, 2014	Poland	Retrospective	DLSS	NR	50/50	51.28 ± 12.08/57.74 ± 9.22	120
2	Austevoll, 2016	Norway	Retrospective	DLSS, DLS	NR	260/260	66.7 ± 10.0/66.3 ± 9.6	12
3	Austevoll, 2021	Norway	RCT	DLS	92/88	133/129	66.0 ± 7.4/66.5 ± 7.9	24
4	Austevoll, 2020	Norway	Prospective	DLS	205/208	285/285	64.6 ± 9.8/64.8 ± 9.2	12
5	Bovonratwet, 2022	America	Retrospective	DLS	46/67	79/109	72.3 ± 11.2/61.7 ± 12.4	12
6	Chan, 2018	America	Retrospective	DLSS	41/211	84/342	69.9 ± 10.5/60.7 ± 11.0	12
7	Chan, 2019	America	Retrospective	DLS	39/40	71/72	72.3 ± 9.7/62.1 ± 10.6	24
8	Dave, 2019	India	Retrospective	DLDH, DLSS	12/5	37/27	48.16 ± 11.12/51.29 ± 12.44	86.21 ± 7.47/79.55 ± 6.21
9	Försth, 2013	Sweden	Retrospective	DLSS	2239/818	4259/1131	70 ± 10/67 ± 10	24
10	Försth, 2016	Sweden	RCT	DLSS, DLS	85/70	120/113	66.6 ± 7.4/67.2 ± 7.9	24
11	Ghogawala, 2016	America	RCT	DLS	27/26	35/31	66.5 ± 8.0/66.7 ± 7.2	48
12	Hua, 2020	China	Retrospective	DLSS	20/48	32/80	56.7 ± 9.1/ 58.8 ± 10.5	24
13	Hua, 2021	China	Retrospective	DLSS, DLS	16/26	24/36	59.0 ± 7.9/59.9 ± 8.6	24
14	Inose, 2022	Japan	RCT	DLS	12/20	29/31	63.4 ± 8.7/63.5 ± 6.8	144
15	Kim, 2015	Korea	Prospective	DLFS	16/21	25/30	73.12 ± 6.75/70.00 ± 5.62	12
16	Kim, 2018	Korea	Prospective	DLS	53/47	68/61	65.47 ± 9.03/66.75 ± 8.77	24
17	Kleinstueck, 2012	Switzerland	Prospective	DLS	33/122	56/157	73.0 ± 8.0/67.4 ± 9.4	12
18	Kuo, 2019	Canada	Retrospective	DLS	105/312	164/437	68.5 ± 9.6/69.2 ± 9.6	60
19	Kurogochi, 2023	Japan	Retrospective	DLS	NR	47/63	71.8 ± 7.8/71.5 ± 6.8	12
20	Lenga, 2024	Germany	Retrospective	DLSS, DLS	170/44	327/89	82.5 ± 2.5/81.7 ± 1.4	36
21	Lin, 2019	China	Retrospective	DLDH	9/4	33/16	57 ± 15.2/51.3 ± 12.4	51.2 ± 33/37.4 ± 19.6
22	Park, 2012	Korea	Retrospective	DLS	15/22	20/25	67.7 ± 7.3/61.9 ± 8.0	54.9 ± 7/69.4 ± 5.25
23	Shafiekhani, 2024	America	Prospective	DLSS	21/21	38/38	53.77 ± 5.069/55.8 ± 8.215	12
24	Shahi, 2024	America	Retrospective	DLS	53/85	87/146	73.0 ± 9.9/63.8 ± 10.4	24
25	Shahi, 2024 (2)	America	Retrospective	DLDH	51/35	120/80	63.34 ± 16.77/53.13 ± 15.33	24
26	Son, 2013	Korea	Retrospective	DLSS	15/18	31/29	72.8 ± 6.8/69.4 ± 3.8	69.6 ± 20.4/62.4 ± 37.2
27	Staartjes, 2018	Switzerland	Prospective	DLS	29/26	51/51	52.7 ± 8.4/53.5 ± 11.1	21.7 ± 4.8
28	Sun, 2014	China	Prospective	DLDH	NR	38/42	30.3 ± 7.3/34.9 ± 4.7	12
29	Thomas, 2019	Canada	Prospective	DLSS	65/57	199/107	65.5 ± 11.6/63.2 ± 9.9	24
30	Tozawa, 2022	Japan	Retrospective	DLS	10/23	18/24	70.4 ± 8.7/70.2 ± 9.1	24
31	Tu, 2021	China	Retrospective	DLSS	9/6	15/13	68.466 ± 4.033/67.307 ± 4.750	12
32	Tye, 2016	America	Retrospective	DLSS	68/46	227/137	49.9 ± 10.3/48.7 ± 8.3	36
33	Ulrich, 2017	Switzerland	Retrospective	DLSS	53/23	85/46	75.4 ± 7.6/68.0 ± 7.8	36
34	Yagi, 2018	Japan	Retrospective	DLS	22/16	59/40	68.5 ± 9.3/66.7 ± 7.1	36
35	Yi, 2020	China	Retrospective	DLSS	105/128	236/261	60.0 ± 16.5/63.0 ± 14.8	24

DA: Decompression alone; DF: Decompression with interbody fusion; RCT: Randomized controlled trial; DLDH: Degenerative lumbar disc herniation; DLSS: Degenerative lumbar spinal stenosis; DLFS: Degenerative lumbar foraminal stenosis; DLS: Degenerative lumbar spondylolisthesis; NR: Not reported.

### Evaluation of the quality of the studies

**Fig 2:**
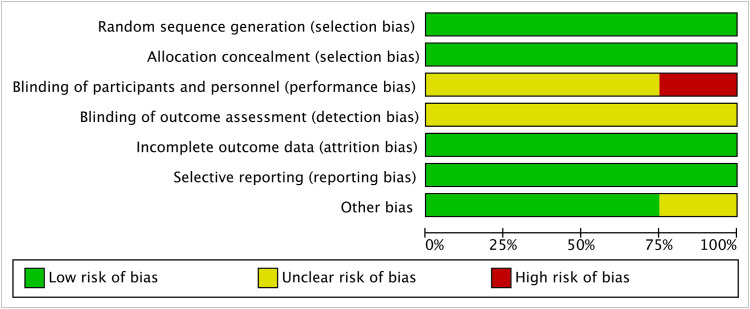
Risk of bias graph.

**Fig 3:**
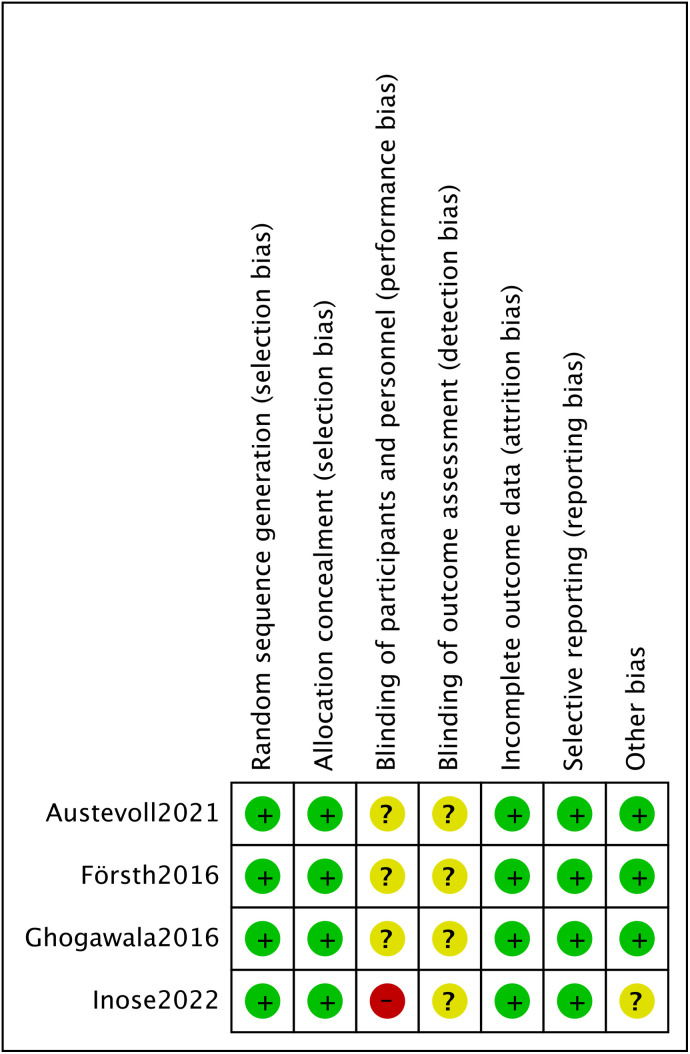
Risk of bias summary.

The literature review encompassed 8 prospective [10,24–26,32,36–38] and 23 retrospective [14,16–22,27–31,33–35,39–45] investigations. Assessment via the NOS classified all studies as possessing moderate to high methodological quality, as detailed in Table 2. Furthermore, 4 RCTs [8,9,15,23] underwent quality appraisal through the Cochrane Collaboration’s instrument within Review Manager version 5.4 (Figs 2 and 3).

**Table 2:** Study evaluation using modified Newcastle-Ottawa Scale.

Author	Study design				Comparability	Exposure			Scores	Quality rating
	Case definition	Case representativeness	Selection of Controls	Definition of Controls	Comparability of cases and controls	Ascertainment of exposure	Same methods of ascertainment for cases and controls	Non-response rate		
Aleksandra, 2014	1	1	1	0	1	1	1	1	7	High quality
Austevoll, 2016	1	1	1	0	2	1	0	1	7	High quality
Austevoll, 2020	1	1	1	1	2	1	1	1	9	High quality
Bovonratwet, 2022	1	1	1	0	2	1	1	1	8	High quality
Chan, 2018	1	1	1	0	1	1	1	1	7	High quality
Chan, 2019	1	1	1	0	2	1	1	0	7	High quality
Dave, 2019	1	1	1	0	2	0	1	1	7	High quality
Försth, 2013	1	1	1	0	2	1	1	1	8	High quality
Hua, 2020	1	1	1	0	2	1	1	1	8	High quality
Hua, 2021	1	1	1	0	2	0	1	1	7	High quality
Kim, 2015	1	1	1	1	2	1	0	1	8	High quality
Kim, 2018	1	1	1	1	1	0	1	1	7	High quality
Kleinstueck, 2012	1	1	1	1	2	0	0	1	7	High quality
Kuo, 2019	1	1	1	0	2	1	1	1	8	High quality
Kurogochi, 2023	1	1	1	0	2	1	0	1	7	High quality
Lenga, 2024	1	1	1	0	2	1	0	1	7	High quality
Lin, 2019	1	1	1	0	1	0	1	1	6	Moderate quality
Park, 2012	1	1	1	0	2	0	1	1	7	High quality
Shafiekhani, 2024	1	1	1	1	2	0	0	1	7	High quality
Shahi, 2024	1	1	1	0	2	1	0	1	7	High quality
Shahi, 2024 (2)	1	1	1	0	2	0	0	1	6	Moderate quality
Son, 2013	1	1	1	0	2	0	1	1	7	High quality
Staartjes, 2018	1	1	1	1	2	0	1	1	8	High quality
Sun, 2014	1	1	1	1	1	1	0	1	7	High quality
Thomas, 2019	1	1	1	1	2	1	1	1	9	High quality
Tozawa, 2022	1	1	1	0	2	0	1	1	7	High quality
Tu, 2021	1	1	1	0	1	0	1	1	6	Moderate quality
Tye, 2016	1	1	1	0	2	1	1	1	8	High quality
Ulrich, 2017	1	1	1	0	1	1	1	1	7	High quality
Yagi, 2018	1	1	1	0	2	0	1	1	7	High quality
Yi, 2020	1	1	1	0	2	0	1	1	7	High quality

### Meta‑analysis

**Fig 4:**
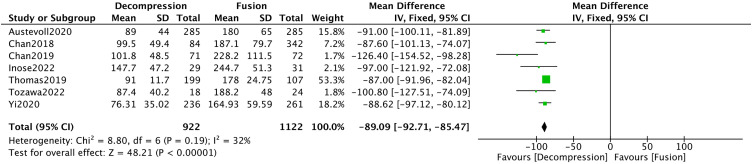
Forest plot of operation time.

Twenty studies [8–10,16–18,21–23,25,29,32,37,38,40,41,45] reported operative durations. The meta-analysis exhibited substantial heterogeneity among these studies (**I*^*2*^* = 98%). After conducting a sensitivity analysis and excluding thirteen studies, this heterogeneity decreased substantially to **I*^*2*^* = 32%. Subsequently, Meta-analysis demonstrated that the DA group had significantly shorter operative duration compared to controls (MD = −89.09, 95%CI −92.71, −85.47, *P* < 0.00001) (Fig 4).

**Fig 5:**
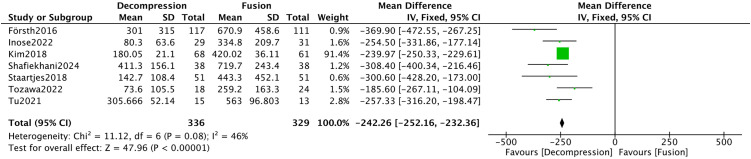
Forest plot of intraoperative blood loss.

Eighteen studies [8,9,16–18,21–23,25,29,32,35–41] submitted intraoperative hemorrhage. The results exhibited significant heterogeneity among the included studies (*I*^*2*^* *= 99%). After conducting a sensitivity analysis and removing eleven studies, the heterogeneity was substantially reduced (*I*^*2*^* *= 46%). Meta-analysis revealed that the DA group experienced significantly less intraoperative hemorrhage (MD = −242.26, 95%CI −252.16, −232.36, *P* < 0.00001) (Fig 5).

**Fig 6:**
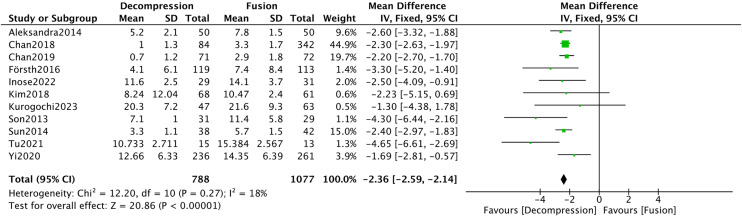
Forest plot of hospital length stay.

Twenty studies [8–10,16–18,21–23,25,28,32,35–41,45] provided data on hospitalization length. The results showed significant heterogeneity among the included studies (*I*^*2*^* *= 97%). After conducting a sensitivity analysis and excluding nine studies, the heterogeneity was substantially reduced (*I*^*2*^* *= 18%). The meta-analysis presented that the DA group had a significantly shorter hospitalization length (MD = −2.36, 95%CI −2.59, −2.14, *P* < 0.00001) (Fig 6).

**Fig 7:**
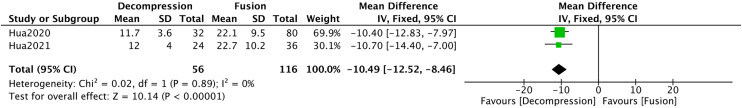
Forest plot of time to ambulation.

Three studies [21,22,40] provided data on the time to ambulation. The results revealed high heterogeneity among the included studies (*I*^*2*^* *= 97%). A sensitivity analysis and the removal of one study significantly reduced this heterogeneity (*I*^*2*^* *= 0%). The meta-analysis presented that the DA group had significantly shorter time to ambulation (MD = −10.49, 95%CI −12.52, −8.46, *P* < 0.00001) (Fig 7).

**Fig 8:**
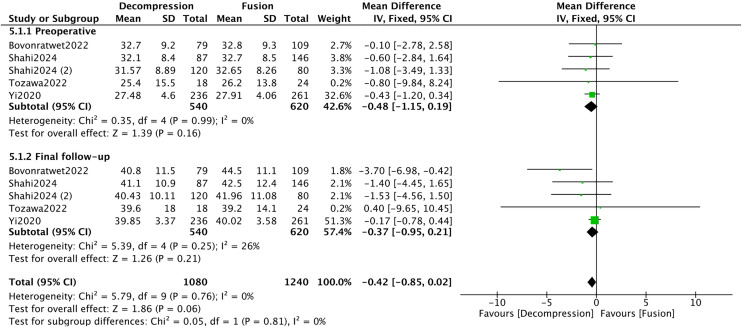
Forest plot of SF12-PCS.

Five studies [16,33,34,39,45] provided both preoperative and final follow-up data for the SF12-PCS. Subgroup analyses revealed low heterogeneity in all groups (*I*^*2 *^< 50%). No significant differences were observed in the SF12-PCS between the DA and DF groups (*P* > 0.05) (Fig 8).

**Fig 9:**
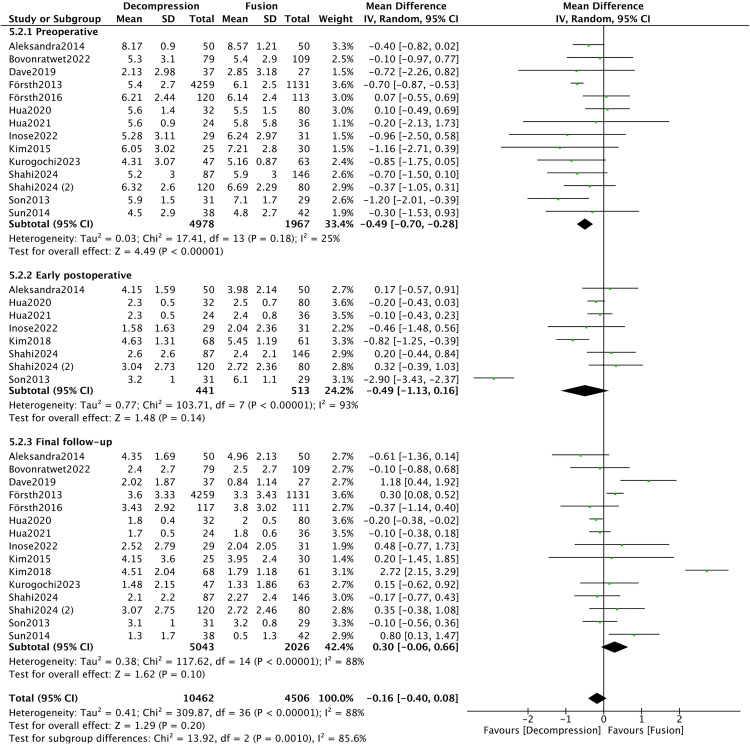
Forest plot of back VAS score.

Fourteen articles [8,16,19–24,28,33–35,37,40] provided data on low back pain VAS score. Among these, fourteen, eight, and fifteen articles reported preoperative, early postoperative, and final follow-up data respectively. A significant difference was found in preoperative low back pain VAS score between the DA and DF groups (MD = −0.49, 95%CI −0.70, −0.28, *P* < 0.00001). Subgroup analysis presented heterogeneity was substantial in the early postoperative and final follow-up subgroups, as well as in the total dataset (*I*^*2 *^> 50%). No significant differences were observed in low back pain VAS score between the DA and DF groups (*P* > 0.05) (Fig 9).

**Fig 10:**
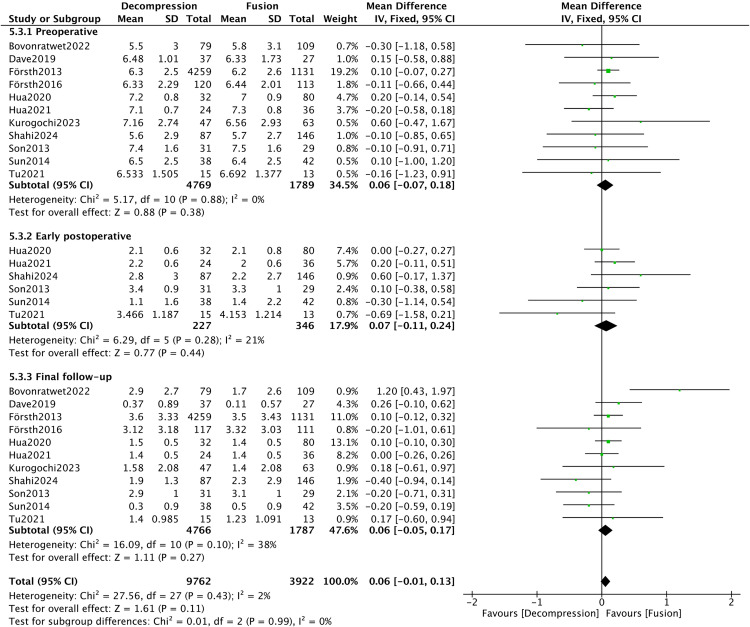
Forest plot of leg VAS score.

A total of eleven studies [8,16,19–22,28,33,35,37,41] provided data on leg pain VAS score, with eleven, six, and eleven studies reporting preoperative, early postoperative, and final follow-up measurements, respectively. The overall heterogeneity was minimal (*I*^*2*^* *= 2%). Subgroup analysis displayed no difference was found in leg pain VAS score between the DA and DF groups (*P* > 0.05) (Fig 10).

**Fig 11:**
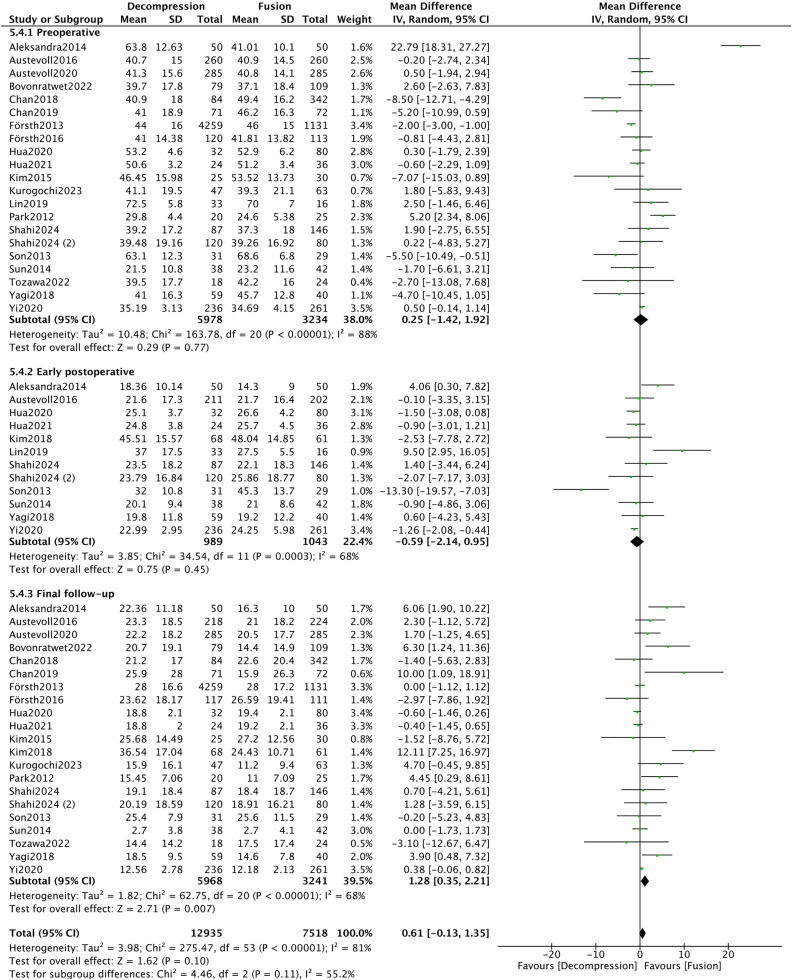
Forest plot of ODI.

Twenty-one articles [8,10,14,16–18,20–22,24,25,28,31,33–35,37,39,40,44,45] offered data on ODI. Specifically, twenty-one, twelve, and twenty-one articles reported preoperative, early postoperative, and final follow-up data, respectively. Total heterogeneity test result was evident (*I*^*2*^* *= 81%). There were no significant differences between the two groups before surgery and early postoperatively (*P* > 0.05). However, the DF group demonstrated statistically superior outcomes compared to the DA group at the final follow-up (MD = 1.28, 95%CI 0.35, 2.21, **P* *= 0.007) (Fig 11).

**Fig 12:**
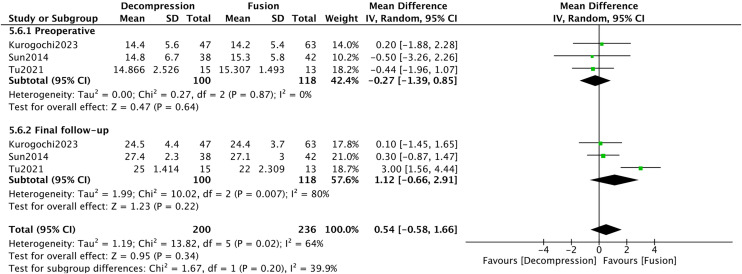
Forest plot of JOA.

Three studies [28,37,41] provided preoperative and final follow-up data for the JOA. The total heterogeneity test result was high (*I*^*2*^* *= 64%). Subgroup analysis revealed no difference was found in JOA between the DA and DF groups (*P* > 0.05) (Fig 12).

**Fig 13:**
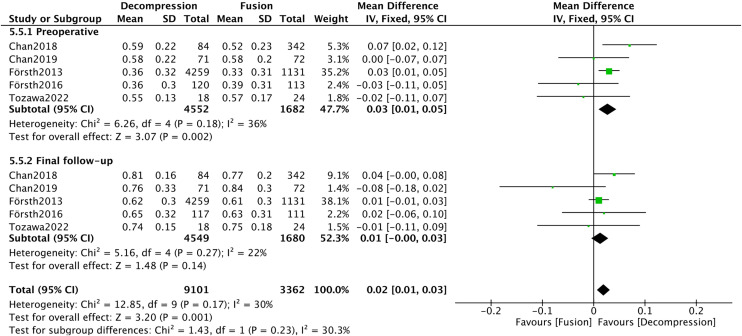
Forest plot of EQ-5D.

Five studies [8,17,18,20,39] provided EQ-5D for both preoperative and final follow-up assessments. A significant difference was evident in the preoperative EQ-5D between the DA and DF groups (MD = 0.03, 95%CI 0.01, 0.05, *Р* = 0. 002), but not in the final follow-up. The total heterogeneity across these studies was minimal (*I*^*2*^* *= 30%). Subgroup analysis further indicated a significant difference in EQ-5D between these groups (MD = 0.02, 95%CI 0.01, 0.03, *Р* = 0.001) (Fig 13). Given the significant preoperative differences, the interpretation of these results requires caution.

**Fig 14:**
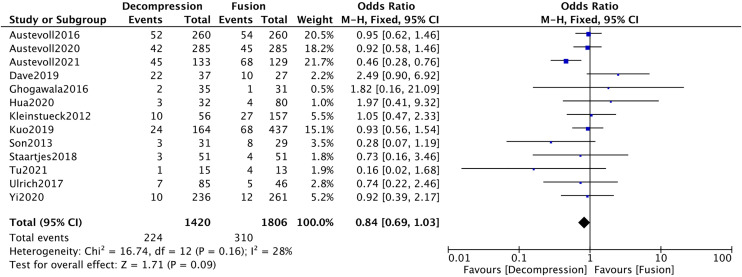
Forest plot of complication rates.

Fourteen studies [9,10,14,15,19,21,26,27,29,35,36,41,43,45] submitted incidence of complications. The results revealed significant heterogeneity among the included studies (*I*^*2*^* *= 63%). After conducting a sensitivity analysis and removing one study, the heterogeneity was substantially reduced (*I*^*2*^* *= 28%). There were no significant differences between the two groups (*P* > 0.05) (Fig 14).

**Fig 15:**
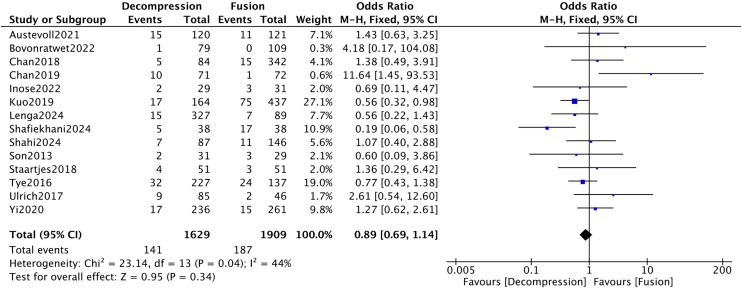
Forest plot of reoperation rates.

Fourteen articles [15–18,23,27,29,32,33,35,36,42,43,45] reported on reoperation rates, enrolling 1,629 and 1,909 patients in two groups respectively. The results indicated found low heterogeneity among the included studies (*I*^*2*^* *= 44%). Meta-analysis revealed no significant difference in reoperation rates between the DA and DF groups (*P* > 0.05) (Fig 15).

**Fig 16:**
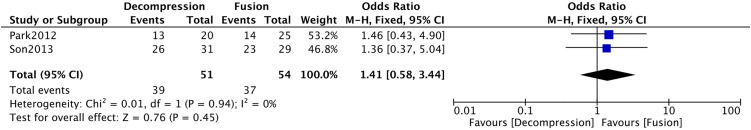
Forest plot of Odom’s criteria.

Three studies [30,31,35] employed Odom’s criteria. The results indicated significant heterogeneity among the included literature (*I*^*2*^* *= 52%). A sensitivity analysis and the exclusion of one study significantly reduced this heterogeneity (*I*^*2*^* *= 0%). Meta-analysis demonstrated no significant difference in Odom’s criteria between the DA and DF groups (*P* > 0.05) (Fig 16).

**Fig 17:**
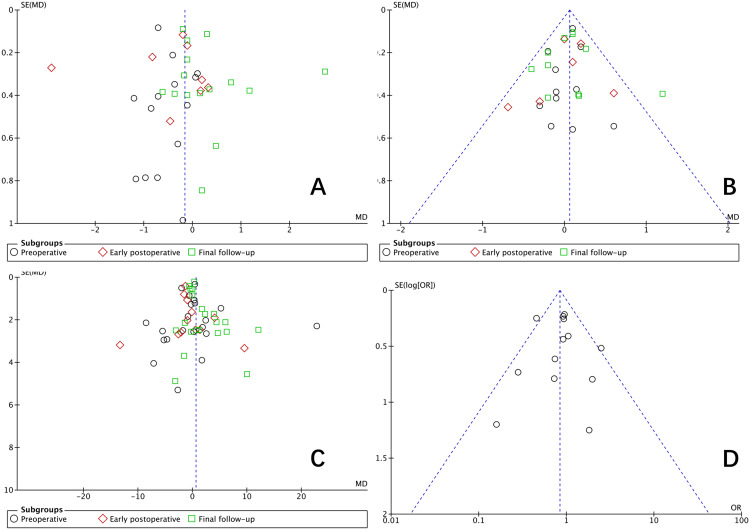
Funnel plots of outcome indicators. **A** low back pain VAS score; **B** leg pain VAS score; **C** ODI; **D** complication rates.

Publication bias was assessed using funnel plots based on low back pain VAS score, leg pain VAS score, ODI, and complication rates as key metrics. The largely symmetrical appearance of the funnel plots suggested a low likelihood of publication bias (Fig 17).

## Discussion

The meta-analysis revealed that the DA group exhibited significantly reduced surgical duration, decreased intraoperative hemorrhage, shorter hospitalization length, and earlier resumption of ambulation compared to the DF group (**P* *< 0.05). Yang et al.‘s study on lumbar spinal stenosis, encompassing 21 RCTs and 3,636 patients, reached similar conclusions [46]. Furthermore, this meta-analysis concluded that there were no significant differences in the results of SF12-PCS, back VAS score, leg VAS score, ODI, JOA, Odom’s criteria, and reoperation rates between the DA and DF groups (*P* > 0.05). The results align with Wu et al.’s study on DLS, which included only RCTs [47]. Another study on DLS indicated that early postoperative low back pain VAS score slightly favored the DF group [48]. However, no significant difference was observed at the final follow-up [48]. Although the EQ-5D showed a slight advantage for the DA group, this was influenced by significant preoperative differences (*P* < 0.05). Postoperative results showed no significant difference (*P* > 0.05). A similar trend in dorsal VAS score suggests potential biases in the included literature. The difference in ODI at the final follow-up was minor and lacked clinical significance. However, it reached statistical significance, possibly attributable to the large sample size.

When DLD is subdivided into different diseases, slight variations in results emerge. Byvaltsev et al. [49] conducted a single-center retrospective study on intervertebral disc disease, finding that the DA group exhibited better early outcomes for ODI, whereas the DF group demonstrated superior results at the final follow-up. Another study involving 29,066 patients with lumbar spinal stenosis reported no significant differences in SF12-PCS, back and leg VAS score or ODI [50]. Kaiser et al.‘s study on DLS showed comparable treatment outcomes and quality of life between the two groups [51]. However, omitting fusion may marginally reduce back pain while slightly increasing the reoperation rate compared to DF. Pranata et al. [52] observed that the DF group exhibited advantages in ODI, back VAS score, and leg VAS score, whereas the DA group performed better in terms of operative time, hospital stay, and intraoperative blood loss. A two-year study on DLS patients found that instability characteristics did not result in superior clinical outcomes for the DF group [53]. Additionally, Gadjradj et al.’s study on lumbar spondylolisthesis with stenosis reported no significant differences in back and leg VAS scores or reoperation rates between the groups [54].

This meta-analysis categorized ODI into preoperative, early postoperative (< 1 year), and final follow-up (≥ 1 year) periods through subgroup analysis. However, heterogeneity remained unchanged, indicating that follow-up time was not the source. To reduce heterogeneity, we suggest improving clinical trial design by standardizing follow-up times. Shen et al. [55] categorized ODI into two subgroups, Caucasians and Mongolians, and concluded that racial differences did not account for the heterogeneity. A study of 6 RCTs and 27 cohort studies with 94,953 patients found no significant difference in ODI at 3, 6, 12, and 24 months [48]. Subgroup analysis of clinical satisfaction showed that trial type (RCT or cohort study) did not impact the results. This meta-analysis used sensitivity analysis to exclude multiple studies for the four outcome indicators: operation time, intraoperative bleeding, hospitalization time, and time to ambulation, aiming to reduce heterogeneity and improve reliability. However, excluding these studies did not affect the outcomes.

Moreover, the findings revealed no significant difference in complication rates between the two groups (**P* *> 0.05). A previous study concluded that there was no significant difference in postoperative complications after treatment for DLSS [55]. A RCT on lumbar spinal stenosis found that the DF group had a higher frequency of proximal adjacent segment stenosis postoperatively but a lower incidence of re-stenosis at the surgical segment [56]. Regardless of preoperative spondylolisthesis, the DA group exhibited increased vertebral slippage postoperatively [56]. A similar study, comprising six RCTs on elderly patients, found a higher rate of lumbar spondylolisthesis progression in the DA group but fewer in-hospital complications and adverse events [57]. A multicenter 5-year RCT on DLS showed a higher dural tear rate in the fusion group [58]. They demonstrated adverse events occurred in 13% of the DA group and 19% of the DF group, with no significant difference. Another study on first-grade spondylolisthesis revealed fewer complications but higher reoperation rate in the DA cohort [59].

Compared to other studies, the advantages of DA—reduced operative time, shorter hospitalization, and less blood loss—are consistently observed, though its efficacy may vary slightly across conditions. Given these benefits, DA appears cost-effective for most patients without specific contraindications. DF may be preferable for DLDH patients presenting with cauda equina syndrome or massive central disc herniation [19,30]. Based on the Michigan State University classification, Li et al. [60] recommended percutaneous endoscopic lumbar discectomy for three DLDH subtypes and transforaminal fusion for four others. Shafiekhani et al. [32] proposed DA for mild to moderate DLSS and DF for severe cases. Shahi et al. [34] recommended DF for patients with spinal instability, isthmic spondylolisthesis, severe disc collapse with foraminal stenosis, the need for realignment, or resection of more than 50% of the facet joint. Similarly, the World Federation of Neurosurgical Societies advised fusion for symptomatic unstable spondylolisthesis and bilateral facet resection exceeding 50% [61]. Two surveys of spine surgeons identified instability, spondylolisthesis grade, lateral translation, hypermobility, mechanical low back pain, and high activity demands as key factors influencing fusion decisions [62,63]. DF may stabilize painful segments and prevent progression in severe dynamic instability or high-risk cases. Dijkerman et al. [64] suggested fusion for high-grade spondylolisthesis or low-grade cases with foraminal stenosis or instability. These findings underscore the importance of assessing spinal stability in both DLS and DLSS. Various posture-based imaging techniques assist in evaluating intervertebral stability [65,66]. In DLS patients with sagittal facet malalignment and significant effusion, fusion may be appropriate [36]. Tools such as the Jakarta Instability Score and the Degenerative Lumbar Spondylolisthesis Instability Classification further support surgical decision-making, particularly for Type III DLS patients with instability who may benefit from fusion [67,68].

The advantages of DF remain indistinct at present, and its overuse has been observed. Our results indicated that DF did not offer additional benefits in treating DLD. Despite its frequent clinical use, DF is sometimes applied unnecessarily. A Canadian prospective study on DLS revealed that DF was used to treat stable, potentially unstable, and unstable cases in 59%, 77%, and 100% of cases, respectively [69]. Data from the American College of Surgeons National Surgical Quality Improvement Program (2005–2015) showed that 90.6% of spondylolisthesis patients opted for fusion surgery [70]. Sastry et al. [71] indicated that the proportion of DF procedures for treating DLSS and DLS in the United States increased from 67.4% in 2016 to 90.4% in 2019, while DA procedures declined from 32.6% to 9.6% over the same period. Kaiser et al. [51] found that not all patients undergoing DF had unstable characteristics, suggesting that 4% to 24% of patients requiring surgery for DLS could be candidates for simple decompression. This highlights the need for clinicians to exercise caution when selecting DF as a surgical option.

### Limitations

The primary limitation lies in the quality and language of the included literature, which is exclusively in English. Additionally, the diverse regions represented may reflect varying technical capabilities. Data analysis is limited by potential biases from high attrition rates in long-term follow-ups and small sample sizes in short-term studies. Sensitivity analysis excluded several articles, which could introduce bias and reduce the sample size. High heterogeneity in the subgroup analysis may impact the results. The follow-up times for subgroup analysis were imprecise, with recent postoperative indicators including markers such as three and six months. Long-term postoperative indicators include markers beyond one year, such as at one, two, and six years. Other limitations include variations due to time and technological advances. This study focused on primary surgeries, excluding secondary surgeries where fusion is commonly used. The question of whether fusion should be the first treatment option for DLS remains controversial. Finally, the inclusion criteria for DLD are too broad, and future studies should focus on more specific disease subtypes.

## Conclusion

Meta-analysis results revealed the DA group and the DF group exhibit comparable clinical effectiveness and patient satisfaction levels. The DA group demonstrated advantages in terms of shorter operative duration, reduced intraoperative hemorrhage, decreased hospitalization length, and quicker time to postoperative ambulation. The difference in ODI at the final follow-up was minor and lacked clinical significance. However, it reached statistical significance, possibly attributable to the large sample size. These findings suggest that DA should be considered the preferred surgical approach for most DLD patients, unless specific indications for fusion exist. Future studies should aim to delineate which patient subsets derive greater benefits from decompression combined with fusion procedures.

## Supporting information

S1 File PRISMA checklist. (DOCX)

S2 File Search strategy. (DOCX)

S3 File Raw data set. (DOCX)
