# Supplementary material for: Decompression with interbody fusion versus decompression alone for degenerative lumbar diseases: A meta-analysis
Source: PLoS One. 2025 Aug 26;20(8):e0330926. doi: 10.1371/journal.pone.0330926 (PMC12380314; doi:10.1371/journal.pone.0330926)
Supplement: S2 File — (DOCX) [file pone.0330926.s002.docx]

**Search strategy**

**Pubmed 2024/07/01——1660 articles**

#1: Intervertebral Disc Degeneration [MeSH Terms]

#2: lumbar disc herniation [Title/Abstract]

#3: lumbar disk herniation [Title/Abstract]

#4: intervertebral disc prolapse [Title/Abstract]

#5: intervertebral disk displacement [Title/Abstract]

#6: lumbar disc protrusion [Title/Abstract]

#7: Spondylolisthesis [MeSH Terms]

#8: Spondylisthesis [Title/Abstract]

#9: Spondylolistheses [Title/Abstract]

#10: Olistheses"[Title/Abstract]

#11: Olisthesis [Title/Abstract]

#12: Spinal Stenosis [MeSH Terms]

#13: spinal stenoses [Title/Abstract]

#14: stenoses spinal [Title/Abstract]

#15: stenosis spinal [Title/Abstract]

#16: #1 OR #2 OR #3 OR #4 OR #5 OR #6 OR #7 OR #8 OR #9 OR #10 OR #11 OR #12 OR #13 OR #14 OR #15

#17: Fusion [Title/Abstract]

#18: Decompression [Title/Abstract]

#19: #16 AND #17 AND #18

**Embase 2024/07/01——2436 articles**

#1: ‘lumbar spinal stenosis’/exp OR ‘lumbar spinal stenosis’

#2: ‘lumbar spinal canal stenosis’:ab,ti

#3: ‘lumbar stenosis’:ab,ti

#4: ‘lumbar spinal stenoses’:ab,ti

#5: ‘lumbar, stenoses, spinal’:ab,ti

#6: ‘lumbar, stenosis, spinal’:ab,ti

#7: ‘spondylolisthesis’ /exp OR ‘spondylolisthesis’

#8: ‘spondylolistheses’:ab,ti

#9: ‘spondylisthesis’:ab,ti

#10: ‘spondylistheses’:ab,ti

#11: ‘olisthesis’:ab,ti

#12: ‘olistheses’:ab,ti

#13: ‘lumbar disc herniation’/exp OR‘lumbar disc herniation’

#14: ‘lumbar disk herniation’:ab,ti

#15: ‘intervertebral disc prolapse’:ab,ti

#16: ‘intervertebral disk displacement’:ab,ti

#17: ‘lumbar disc protrusion’:ab,ti

#18: ‘lumbar slipped disk’:ab,ti

#19: #1 OR #2 OR #3 OR #4 OR #5 OR #6 OR #7 OR #8 OR #9 OR #10 OR #11 OR #12 OR #13 OR #14 OR #15 OR #16 OR #17 OR #18

#20: ‘fusion’:ab,ti

#21: ‘decompression’:ab,ti

#22: #19 AND #20 AND #21

**Web of Science 2024/07/01——3959 articles**

#1: TS=(lumbar spinal canal stenosis OR lumbar spinal stenosis OR lumbar stenosis OR lumbar spinal stenoses OR lumbar, stenoses, spinal OR lumbar, stenosis, spinal OR spondylolisthesis OR Spondylolistheses OR Spondylisthesis OR Spondylistheses OR Olisthesis OR Olistheses OR lumbar disc herniation OR lumbar disk herniation OR intervertebral disc prolapse OR intervertebral disk displacement OR lumbar disc protrusion OR lumbar slipped disk)

#2: TS=(decompression AND fusion)

#3: #1 AND #2
