# Supplementary material for: Decompression with interbody fusion versus decompression alone for degenerative lumbar diseases: A meta-analysis
Source: PLoS One. 2025 Aug 26;20(8):e0330926. doi: 10.1371/journal.pone.0330926 (PMC12380314; doi:10.1371/journal.pone.0330926)
Supplement: S3 File — (DOCX) [file pone.0330926.s003.docx]

**Table 1. Characteristics of the included literature.**

| **NO** | **Author (year)** | **Country** | **Study Design** | **Disease type** | **DA/DF** | | | |
| --- | --- | --- | --- | --- | --- | --- | --- | --- |
|  |  |  |  |  | **Females (N)** | **Gender (N)** | **Age (years)** | **Follow-up (months)** |
| 1 | Aleksandra, 2014 | Poland | Retrospective | DLSS | NR | 50/50 | 51.28±12.08/57.74±9.22 | 120 |
| 2 | Austevoll, 2016 | Norway | Retrospective | DLSS, DLS | NR | 260/260 | 66.7±10.0/66.3±9.6 | 12 |
| 3 | Austevoll, 2021 | Norway | RCT | DLS | 92/88 | 133/129 | 66.0±7.4/66.5±7.9 | 24 |
| 4 | Austevoll, 2020 | Norway | Prospective | DLS | 205/208 | 285/285 | 64.6±9.8/64.8±9.2 | 12 |
| 5 | Bovonratwet, 2022 | America | Retrospective | DLS | 46/67 | 79/109 | 72.3±11.2/61.7±12.4 | 12 |
| 6 | Chan, 2018 | America | Retrospective | DLSS | 41/211 | 84/342 | 69.9±10.5/60.7±11.0 | 12 |
| 7 | Chan, 2019 | America | Retrospective | DLS | 39/40 | 71/72 | 72.3±9.7/62.1±10.6 | 24 |
| 8 | Dave, 2019 | India | Retrospective | DLDH, DLSS | 12/5 | 37/27 | 48.16±11.12/51.29±12.44 | 86.21±7.47/79.55±6.21 |
| 9 | Försth, 2013 | Sweden | Retrospective | DLSS | 2239/818 | 4259/1131 | 70±10/67±10 | 24 |
| 10 | Försth, 2016 | Sweden | RCT | DLSS, DLS | 85/70 | 120/113 | 66.6±7.4/67.2±7.9 | 24 |
| 11 | Ghogawala, 2016 | America | RCT | DLS | 27/26 | 35/31 | 66.5±8.0/66.7±7.2 | 48 |
| 12 | Hua, 2020 | China | Retrospective | DLSS | 20/48 | 32/80 | 56.7±9.1/ 58.8±10.5 | 24 |
| 13 | Hua, 2021 | China | Retrospective | DLSS, DLS | 16/26 | 24/36 | 59.0±7.9/59.9±8.6 | 24 |
| 14 | Inose, 2022 | Japan | RCT | DLS | 12/20 | 29/31 | 63.4±8.7/63.5±6.8 | 144 |
| 15 | Kim, 2015 | Korea | Prospective | DLFS | 16/21 | 25/30 | 73.12±6.75/70.00±5.62 | 12 |
| 16 | Kim, 2018 | Korea | Prospective | DLS | 53/47 | 68/61 | 65.47±9.03/66.75±8.77 | 24 |
| 17 | Kleinstueck, 2012 | Switzerland | Prospective | DLS | 33/122 | 56/157 | 73.0±8.0/67.4±9.4 | 12 |
| 18 | Kuo, 2019 | Canada | Retrospective | DLS | 105/312 | 164/437 | 68.5±9.6/69.2±9.6 | 60 |
| 19 | Kurogochi, 2023 | Japan | Retrospective | DLS | NR | 47/63 | 71.8±7.8/71.5±6.8 | 12 |
| 20 | Lenga, 2024 | Germany | Retrospective | DLSS, DLS | 170/44 | 327/89 | 82.5±2.5/81.7±1.4 | 36 |
| 21 | Lin, 2019 | China | Retrospective | DLDH | 9/4 | 33/16 | 57±15.2/51.3±12.4 | 51.2±33/37.4±19.6 |
| 22 | Park, 2012 | Korea | Retrospective | DLS | 15/22 | 20/25 | 67.7±7.3/61.9±8.0 | 54.9±7/69.4±5.25 |
| 23 | Shafiekhani, 2024 | America | Prospective | DLSS | 21/21 | 38/38 | 53.77±5.069/55.8±8.215 | 12 |
| 24 | Shahi, 2024 | America | Retrospective | DLS | 53/85 | 87/146 | 73.0±9.9/63.8±10.4 | 24 |
| 25 | Shahi, 2024 (2) | America | Retrospective | DLDH | 51/35 | 120/80 | 63.34±16.77/53.13±15.33 | 24 |
| 26 | Son, 2013 | Korea | Retrospective | DLSS | 15/18 | 31/29 | 72.8±6.8/69.4±3.8 | 69.6 ± 20.4/62.4 ± 37.2 |
| 27 | Staartjes, 2018 | Switzerland | Prospective | DLS | 29/26 | 51/51 | 52.7±8.4/53.5±11.1 | 21.7 ±4.8 |
| 28 | Sun, 2014 | China | Prospective | DLDH | NR | 38/42 | 30.3±7.3/34.9±4.7 | 12 |
| 29 | Thomas, 2019 | Canada | Prospective | DLSS | 65/57 | 199/107 | 65.5±11.6/63.2±9.9 | 24 |
| 30 | Tozawa, 2022 | Japan | Retrospective | DLS | 10/23 | 18/24 | 70.4±8.7/70.2±9.1 | 24 |
| 31 | Tu, 2021 | China | Retrospective | DLSS | 9/6 | 15/13 | 68.466±4.033/67.307±4.750 | 12 |
| 32 | Tye, 2016 | America | Retrospective | DLSS | 68/46 | 227/137 | 49.9±10.3/48.7±8.3 | 36 |
| 33 | Ulrich, 2017 | Switzerland | Retrospective | DLSS | 53/23 | 85/46 | 75.4±7.6/68.0±7.8 | 36 |
| 34 | Yagi, 2018 | Japan | Retrospective | DLS | 22/16 | 59/40 | 68.5±9.3/66.7±7.1 | 36 |
| 35 | Yi, 2020 | China | Retrospective | DLSS | 105/128 | 236/261 | 60.0±16.5/63.0±14.8 | 24 |

DA: Decompression alone; DF: Decompression with interbody fusion; RCT: Randomized controlled trial; DLDH: Degenerative lumbar disc herniation; DLSS: Degenerative lumbar spinal stenosis; DLFS: Degenerative lumbar foraminal stenosis; DLS: Degenerative lumbar

spondylolisthesis; NR: Not reported.

**Table 2. Study Evaluation Using Modified Newcastle-Ottawa Scale**

| **Author** | **Study design** | | | | **Comparability** | **Exposure** | | | **Scores** | **Quality rating** |
| --- | --- | --- | --- | --- | --- | --- | --- | --- | --- | --- |
|  | **Case  definition** | **Case  representativeness** | **Selection  of  Controls** | **Definition  of  Controls** | **Comparability  of cases and  controls** | **Ascertainment  of exposure** | **Same  methods of  ascertainment  for cases and  controls** | **Non-response  rate** |  |  |
| Aleksandra, 2014 | 1 | 1 | 1 | 0 | 1 | 1 | 1 | 1 | 7 | High quality |
| Austevoll, 2016 | 1 | 1 | 1 | 0 | 2 | 1 | 0 | 1 | 7 | High quality |
| Austevoll2020 | 1 | 1 | 1 | 1 | 2 | 1 | 1 | 1 | 9 | High quality |
| Bovonratwet, 2022 | 1 | 1 | 1 | 0 | 2 | 1 | 1 | 1 | 8 | High quality |
| Chan, 2018 | 1 | 1 | 1 | 0 | 1 | 1 | 1 | 1 | 7 | High quality |
| Chan, 2019 | 1 | 1 | 1 | 0 | 2 | 1 | 1 | 0 | 7 | High quality |
| Dave, 2019 | 1 | 1 | 1 | 0 | 2 | 0 | 1 | 1 | 7 | High quality |
| Försth, 2013 | 1 | 1 | 1 | 0 | 2 | 1 | 1 | 1 | 8 | High quality |
| Hua, 2020 | 1 | 1 | 1 | 0 | 2 | 1 | 1 | 1 | 8 | High quality |
| Hua, 2021 | 1 | 1 | 1 | 0 | 2 | 0 | 1 | 1 | 7 | High quality |
| Kim, 2015 | 1 | 1 | 1 | 1 | 2 | 1 | 0 | 1 | 8 | High quality |
| Kim, 2018 | 1 | 1 | 1 | 1 | 1 | 0 | 1 | 1 | 7 | High quality |
| Kleinstueck, 2012 | 1 | 1 | 1 | 1 | 2 | 0 | 0 | 1 | 7 | High quality |
| Kuo, 2019 | 1 | 1 | 1 | 0 | 2 | 1 | 1 | 1 | 8 | High quality |
| Kurogochi, 2023 | 1 | 1 | 1 | 0 | 2 | 1 | 0 | 1 | 7 | High quality |
| Lenga, 2024 | 1 | 1 | 1 | 0 | 2 | 1 | 0 | 1 | 7 | High quality |
| Lin, 2019 | 1 | 1 | 1 | 0 | 1 | 0 | 1 | 1 | 6 | Moderate quality |
| Park, 2012 | 1 | 1 | 1 | 0 | 2 | 0 | 1 | 1 | 7 | High quality |
| Shafiekhani, 2024 | 1 | 1 | 1 | 1 | 2 | 0 | 0 | 1 | 7 | High quality |
| Shahi, 2024 | 1 | 1 | 1 | 0 | 2 | 1 | 0 | 1 | 7 | High quality |
| Shahi, 2024 (2) | 1 | 1 | 1 | 0 | 2 | 0 | 0 | 1 | 6 | Moderate quality |
| Son, 2013 | 1 | 1 | 1 | 0 | 2 | 0 | 1 | 1 | 7 | High quality |
| Staartjes, 2018 | 1 | 1 | 1 | 1 | 2 | 0 | 1 | 1 | 8 | High quality |
| Sun, 2014 | 1 | 1 | 1 | 1 | 1 | 1 | 0 | 1 | 7 | High quality |
| Thomas, 2019 | 1 | 1 | 1 | 1 | 2 | 1 | 1 | 1 | 9 | High quality |
| Tozawa, 2022 | 1 | 1 | 1 | 0 | 2 | 0 | 1 | 1 | 7 | High quality |
| Tu, 2021 | 1 | 1 | 1 | 0 | 1 | 0 | 1 | 1 | 6 | Moderate quality |
| Tye, 2016 | 1 | 1 | 1 | 0 | 2 | 1 | 1 | 1 | 8 | High quality |
| Ulrich, 2017 | 1 | 1 | 1 | 0 | 1 | 1 | 1 | 1 | 7 | High quality |
| Yagi, 2018 | 1 | 1 | 1 | 0 | 2 | 0 | 1 | 1 | 7 | High quality |
| Yi, 2020 | 1 | 1 | 1 | 0 | 2 | 0 | 1 | 1 | 7 | High quality |

| Funnel plot of operation time |  |  |  |  |  |  |
| --- | --- | --- | --- | --- | --- | --- |
|  | D group |  |  | F group |  |  |
| Author | mean | sd | total | mean | sd | total |
| Aleksandra2014 | 41 | 3.2 | 50 | 82 | 11.4 | 50 |
| Austevoll2020 | 89 | 44 | 285 | 180 | 65 | 285 |
| Bovonratwet2022 | 81.4 | 40.7 | 79 | 111.4 | 52.9 | 109 |
| Chan2018 | 99.5 | 49.4 | 84 | 187.1 | 79.7 | 342 |
| Chan2019 | 101.8 | 48.5 | 71 | 228.2 | 111.5 | 72 |
| Försth2016 | 88.5 | 35.9 | 117 | 149.4 | 45 | 111 |
| Ghogawala2016 | 124.4 | 34.2 | 35 | 289.6 | 66.3 | 31 |
| Hua2020 | 139.5 | 31.2 | 32 | 161.1 | 45.6 | 80 |
| Hua2021 | 142.5 | 34.2 | 24 | 158 | 42.8 | 36 |
| Inose2022 | 147.7 | 47.2 | 29 | 244.7 | 51.3 | 31 |
| Kim2018 | 55.09 | 12.16 | 68 | 78.35 | 11.92 | 61 |
| Lenga2024 | 145 | 50.2 | 327 | 290.3 | 106.8 | 89 |
| Shafiekhani2024 | 167.52 | 49.23 | 38 | 418.08 | 56.8 | 38 |
| Son2013 | 138 | 36 | 31 | 312 | 108 | 29 |
| Staartjes2018 | 25.7 | 11.2 | 51 | 161 | 45.9 | 51 |
| Sun2014 | 56.4 | 14.5 | 38 | 124.5 | 27.2 | 42 |
| Thomas2019 | 91 | 11.7 | 199 | 178 | 24.75 | 107 |
| Tozawa2022 | 87.4 | 40.2 | 18 | 188.2 | 48 | 24 |
| Tu2021 | 107.8 | 15.335 | 15 | 159.153 | 19.582 | 13 |
| Yi2020 | 76.31 | 35.02 | 236 | 164.93 | 59.59 | 261 |

| Funnel plot of intraoperative blood loss |  |  |  |  |  |  |
| --- | --- | --- | --- | --- | --- | --- |
|  | D group |  |  | F group |  |  |
| Author | mean | sd | total | mean | sd | total |
| Aleksandra2014 | 110 | 25.1 | 50 | 205 | 30.7 | 50 |
| Bovonratwet2022 | 23.9 | 12.2 | 79 | 48.9 | 33.7 | 109 |
| Chan2018 | 44.5 | 59 | 84 | 230.1 | 203.8 | 342 |
| Chan2019 | 33 | 63.7 | 71 | 108.8 | 85.6 | 72 |
| Försth2016 | 301 | 315 | 117 | 670.9 | 458.6 | 111 |
| Ghogawala2016 | 83.4 | 63.5 | 35 | 513.7 | 334.4 | 31 |
| Hua2020 | 51.9 | 10.9 | 32 | 146.6 | 80.3 | 80 |
| Hua2021 | 50.4 | 10.8 | 24 | 149.4 | 89.8 | 36 |
| Inose2022 | 80.3 | 63.6 | 29 | 334.8 | 209.7 | 31 |
| Kim2018 | 180.05 | 21.1 | 68 | 420.02 | 36.11 | 61 |
| Lenga2024 | 336.1 | 150.8 | 327 | 791.6 | 319.3 | 89 |
| Shafiekhani2024 | 411.3 | 156.1 | 38 | 719.7 | 243.4 | 38 |
| Son2013 | 100 | 20 | 31 | 560 | 210 | 29 |
| Staartjes2018 | 142.7 | 108.4 | 51 | 443.3 | 452.1 | 51 |
| Sun2014 | 31 | 18.1 | 38 | 331.2 | 137.3 | 42 |
| Thomas2019 | 75 | 37.5 | 199 | 400 | 117.2 | 107 |
| Tozawa2022 | 73.6 | 105.5 | 18 | 259.2 | 163.3 | 24 |
| Tu2021 | 305.666 | 52.14 | 15 | 563 | 96.803 | 13 |

| Funnel plot of hospital length stay |  |  |  |  |  |  |
| --- | --- | --- | --- | --- | --- | --- |
|  | D group |  |  | F group |  |  |
| Author | mean | sd | total | mean | sd | total |
| Aleksandra2014 | 5.2 | 2.1 | 50 | 7.8 | 1.5 | 50 |
| Austevoll2020 | 2.5 | 2.4 | 285 | 6.4 | 3 | 285 |
| Bovonratwet2022 | 19.2 | 25.1 | 79 | 36.4 | 24.5 | 109 |
| Chan2018 | 1 | 1.3 | 84 | 3.3 | 1.7 | 342 |
| Chan2019 | 0.7 | 1.2 | 71 | 2.9 | 1.8 | 72 |
| Försth2016 | 4.1 | 6.1 | 119 | 7.4 | 8.4 | 113 |
| Ghogawala2016 | 2.6 | 0.9 | 35 | 4.2 | 0.9 | 31 |
| Hua2020 | 2.7 | 0.9 | 32 | 11.2 | 2.4 | 80 |
| Hua2021 | 2.6 | 1 | 24 | 11.1 | 2.6 | 36 |
| Inose2022 | 11.6 | 2.5 | 29 | 14.1 | 3.7 | 31 |
| Kim2018 | 8.24 | 12.04 | 68 | 10.47 | 2.4 | 61 |
| Kurogochi2023 | 20.3 | 7.2 | 47 | 21.6 | 9.3 | 63 |
| Shafiekhani2024 | 3.551 | 0.6349 | 38 | 6.774 | 1.197 | 38 |
| Son2013 | 7.1 | 1 | 31 | 11.4 | 5.8 | 29 |
| Staartjes2018 | 28.5 | 13.2 | 51 | 54 | 15 | 51 |
| Sun2014 | 3.3 | 1.1 | 38 | 5.7 | 1.5 | 42 |
| Thomas2019 | 1 | 0.75 | 199 | 4 | 0.5 | 107 |
| Tozawa2022 | 8.1 | 3 | 18 | 16.8 | 8.9 | 24 |
| Tu2021 | 10.733 | 2.711 | 15 | 15.384 | 2.567 | 13 |
| Yi2020 | 12.66 | 6.33 | 236 | 14.35 | 6.39 | 261 |

| Funnel plot of time to ambulation |  |  |  |  |  |  |
| --- | --- | --- | --- | --- | --- | --- |
|  | D group |  |  | F group |  |  |
| Author | mean | sd | total | mean | sd | total |
| Aleksandra2014 | 3.2 | 1 | 50 | 5.5 | 1.7 | 50 |
| Hua2020 | 11.7 | 3.6 | 32 | 22.1 | 9.5 | 80 |
| Hua2021 | 12 | 4 | 24 | 22.7 | 10.2 | 36 |

| Funnel plot of SF12-PCS |  |  |  |  |  |  |  |
| --- | --- | --- | --- | --- | --- | --- | --- |
|  |  | D group |  |  | F group |  |  |
|  | Author | mean | sd | total | mean | sd | total |
| Preoperative | Bovonratwet2022 | 32.7 | 9.2 | 79 | 32.8 | 9.3 | 109 |
|  | Shahi2024 | 32.1 | 8.4 | 87 | 32.7 | 8.5 | 146 |
|  | Shahi2024 (2) | 31.57 | 8.89 | 120 | 32.65 | 8.26 | 80 |
|  | Tozawa2022 | 25.4 | 15.5 | 18 | 26.2 | 13.8 | 24 |
|  | Yi2020 | 27.48 | 4.6 | 236 | 27.91 | 4.06 | 261 |
| Final follow-up | Bovonratwet2022 | 40.8 | 11.5 | 79 | 44.5 | 11.1 | 109 |
|  | Shahi2024 | 41.1 | 10.9 | 87 | 42.5 | 12.4 | 146 |
|  | Shahi2024 (2) | 40.43 | 10.11 | 120 | 41.96 | 11.08 | 80 |
|  | Tozawa2022 | 39.6 | 18 | 18 | 39.2 | 14.1 | 24 |
|  | Yi2020 | 39.85 | 3.37 | 236 | 40.02 | 3.58 | 261 |

| Funnel plot of back VAS |  |  |  |  |  |  |  |
| --- | --- | --- | --- | --- | --- | --- | --- |
|  |  | D group |  |  | F group |  |  |
|  | Author | mean | sd | total | mean | sd | total |
| Preoperative | Aleksandra2014 | 8.17 | 0.9 | 50 | 8.57 | 1.21 | 50 |
|  | Bovonratwet2022 | 5.3 | 3.1 | 79 | 5.4 | 2.9 | 109 |
|  | Dave2019 | 2.13 | 2.98 | 37 | 2.85 | 3.18 | 27 |
|  | Försth2013 | 5.4 | 2.7 | 4259 | 6.1 | 2.5 | 1131 |
|  | Försth2016 | 6.21 | 2.44 | 120 | 6.14 | 2.4 | 113 |
|  | Hua2020 | 5.6 | 1.4 | 32 | 5.5 | 1.5 | 80 |
|  | Hua2021 | 5.6 | 0.9 | 24 | 5.8 | 5.8 | 36 |
|  | Inose2022 | 5.28 | 3.11 | 29 | 6.24 | 2.97 | 31 |
|  | Kim2015 | 6.05 | 3.02 | 25 | 7.21 | 2.8 | 30 |
|  | Kurogochi2023 | 4.31 | 3.07 | 47 | 5.16 | 0.87 | 63 |
|  | Shahi2024 | 5.2 | 3 | 87 | 5.9 | 3 | 146 |
|  | Shahi2024 (2) | 6.32 | 2.6 | 120 | 6.69 | 2.29 | 80 |
|  | Son2013 | 5.9 | 1.5 | 31 | 7.1 | 1.7 | 29 |
|  | Sun2014 | 4.5 | 2.9 | 38 | 4.8 | 2.7 | 42 |
| Early postoperative | Aleksandra2014 | 4.15 | 1.59 | 50 | 3.98 | 2.14 | 50 |
|  | Hua2020 | 2.3 | 0.5 | 32 | 2.5 | 0.7 | 80 |
|  | Hua2021 | 2.3 | 0.5 | 24 | 2.4 | 0.8 | 36 |
|  | Inose2022 | 1.58 | 1.63 | 29 | 2.04 | 2.36 | 31 |
|  | Kim2018 | 4.63 | 1.31 | 68 | 5.45 | 1.19 | 61 |
|  | Shahi2024 | 2.6 | 2.6 | 87 | 2.4 | 2.1 | 146 |
|  | Shahi2024 (2) | 3.04 | 2.73 | 120 | 2.72 | 2.36 | 80 |
|  | Son2013 | 3.2 | 1 | 31 | 6.1 | 1.1 | 29 |
| Final follow-up | Aleksandra2014 | 4.35 | 1.69 | 50 | 4.96 | 2.13 | 50 |
|  | Bovonratwet2022 | 2.4 | 2.7 | 79 | 2.5 | 2.7 | 109 |
|  | Dave2019 | 2.02 | 1.87 | 37 | 0.84 | 1.14 | 27 |
|  | Försth2013 | 3.6 | 3.33 | 4259 | 3.3 | 3.43 | 1131 |
|  | Försth2016 | 3.43 | 2.92 | 117 | 3.8 | 3.02 | 111 |
|  | Hua2020 | 1.8 | 0.4 | 32 | 2 | 0.5 | 80 |
|  | Hua2021 | 1.7 | 0.5 | 24 | 1.8 | 0.6 | 36 |
|  | Inose2022 | 2.52 | 2.79 | 29 | 2.04 | 2.05 | 31 |
|  | Kim2015 | 4.15 | 3.6 | 25 | 3.95 | 2.4 | 30 |
|  | Kim2018 | 4.51 | 2.04 | 68 | 1.79 | 1.18 | 61 |
|  | Kurogochi2023 | 1.48 | 2.15 | 47 | 1.33 | 1.86 | 63 |
|  | Shahi2024 | 2.1 | 2.2 | 87 | 2.27 | 2.4 | 146 |
|  | Shahi2024 (2) | 3.07 | 2.75 | 120 | 2.72 | 2.46 | 80 |
|  | Son2013 | 3.1 | 1 | 31 | 3.2 | 0.8 | 29 |
|  | Sun2014 | 1.3 | 1.7 | 38 | 0.5 | 1.3 | 42 |

| Funnel plot of leg VAS |  |  |  |  |  |  |  |
| --- | --- | --- | --- | --- | --- | --- | --- |
|  |  | D group |  |  | F group |  |  |
|  | Author | mean | sd | total | mean | sd | total |
| Preoperative | Bovonratwet2022 | 5.5 | 3 | 79 | 5.8 | 3.1 | 109 |
|  | Dave2019 | 6.48 | 1.01 | 37 | 6.33 | 1.73 | 27 |
|  | Försth2013 | 6.3 | 2.5 | 4259 | 6.2 | 2.6 | 1131 |
|  | Försth2016 | 6.33 | 2.29 | 120 | 6.44 | 2.01 | 113 |
|  | Hua2020 | 7.2 | 0.8 | 32 | 7 | 0.9 | 80 |
|  | Hua2021 | 7.1 | 0.7 | 24 | 7.3 | 0.8 | 36 |
|  | Kurogochi2023 | 7.16 | 2.74 | 47 | 6.56 | 2.93 | 63 |
|  | Shahi2024 | 5.6 | 2.9 | 87 | 5.7 | 2.7 | 146 |
|  | Son2013 | 7.4 | 1.6 | 31 | 7.5 | 1.6 | 29 |
|  | Sun2014 | 6.5 | 2.5 | 38 | 6.4 | 2.5 | 42 |
|  | Tu2021 | 6.533 | 1.505 | 15 | 6.692 | 1.377 | 13 |
| Early postoperative | Hua2020 | 2.1 | 0.6 | 32 | 2.1 | 0.8 | 80 |
|  | Hua2021 | 2.2 | 0.6 | 24 | 2 | 0.6 | 36 |
|  | Shahi2024 | 2.8 | 3 | 87 | 2.2 | 2.7 | 146 |
|  | Son2013 | 3.4 | 0.9 | 31 | 3.3 | 1 | 29 |
|  | Sun2014 | 1.1 | 1.6 | 38 | 1.4 | 2.2 | 42 |
|  | Tu2021 | 3.466 | 1.187 | 15 | 4.153 | 1.214 | 13 |
| Final follow-up | Bovonratwet2022 | 2.9 | 2.7 | 79 | 1.7 | 2.6 | 109 |
|  | Dave2019 | 0.37 | 0.89 | 37 | 0.11 | 0.57 | 27 |
|  | Försth2013 | 3.6 | 3.33 | 4259 | 3.5 | 3.43 | 1131 |
|  | Försth2016 | 3.12 | 3.18 | 117 | 3.32 | 3.03 | 111 |
|  | Hua2020 | 1.5 | 0.5 | 32 | 1.4 | 0.5 | 80 |
|  | Hua2021 | 1.4 | 0.5 | 24 | 1.4 | 0.5 | 36 |
|  | Kurogochi2023 | 1.58 | 2.08 | 47 | 1.4 | 2.08 | 63 |
|  | Shahi2024 | 1.9 | 1.3 | 87 | 2.3 | 2.9 | 146 |
|  | Son2013 | 2.9 | 1 | 31 | 3.1 | 1 | 29 |
|  | Sun2014 | 0.3 | 0.9 | 38 | 0.5 | 0.9 | 42 |
|  | Tu2021 | 1.4 | 0.985 | 15 | 1.23 | 1.091 | 13 |

| Funnel plot of ODI |  |  |  |  |  |  |  |
| --- | --- | --- | --- | --- | --- | --- | --- |
|  |  | D group |  |  | F group |  |  |
|  | Author | mean | sd | total | mean | sd | total |
| Preoperative | Aleksandra2014 | 63.8 | 12.63 | 50 | 41.01 | 10.1 | 50 |
|  | Austevoll2016 | 40.7 | 15 | 260 | 40.9 | 14.5 | 260 |
|  | Austevoll2020 | 41.3 | 15.6 | 285 | 40.8 | 14.1 | 285 |
|  | Bovonratwet2022 | 39.7 | 17.8 | 79 | 37.1 | 18.4 | 109 |
|  | Chan2018 | 40.9 | 18 | 84 | 49.4 | 16.2 | 342 |
|  | Chan2019 | 41 | 18.9 | 71 | 46.2 | 16.3 | 72 |
|  | Försth2013 | 44 | 16 | 4259 | 46 | 15 | 1131 |
|  | Försth2016 | 41 | 14.38 | 120 | 41.81 | 13.82 | 113 |
|  | Hua2020 | 53.2 | 4.6 | 32 | 52.9 | 6.2 | 80 |
|  | Hua2021 | 50.6 | 3.2 | 24 | 51.2 | 3.4 | 36 |
|  | Kim2015 | 46.45 | 15.98 | 25 | 53.52 | 13.73 | 30 |
|  | Kurogochi2023 | 41.1 | 19.5 | 47 | 39.3 | 21.1 | 63 |
|  | Lin2019 | 72.5 | 5.8 | 33 | 70 | 7 | 16 |
|  | Park2012 | 29.8 | 4.4 | 20 | 24.6 | 5.38 | 25 |
|  | Shahi2024 | 39.2 | 17.2 | 87 | 37.3 | 18 | 146 |
|  | Shahi2024 (2) | 39.48 | 19.16 | 120 | 39.26 | 16.92 | 80 |
|  | Son2013 | 63.1 | 12.3 | 31 | 68.6 | 6.8 | 29 |
|  | Sun2014 | 21.5 | 10.8 | 38 | 23.2 | 11.6 | 42 |
|  | Tozawa2022 | 39.5 | 17.7 | 18 | 42.2 | 16 | 24 |
|  | Yagi2018 | 41 | 16.3 | 59 | 45.7 | 12.8 | 40 |
|  | Yi2020 | 35.19 | 3.13 | 236 | 34.69 | 4.15 | 261 |
| Early postoperative | Aleksandra2014 | 18.36 | 10.14 | 50 | 14.3 | 9 | 50 |
|  | Austevoll2016 | 21.6 | 17.3 | 211 | 21.7 | 16.4 | 202 |
|  | Hua2020 | 25.1 | 3.7 | 32 | 26.6 | 4.2 | 80 |
|  | Hua2021 | 24.8 | 3.8 | 24 | 25.7 | 4.5 | 36 |
|  | Kim2018 | 45.51 | 15.57 | 68 | 48.04 | 14.85 | 61 |
|  | Lin2019 | 37 | 17.5 | 33 | 27.5 | 5.5 | 16 |
|  | Shahi2024 | 23.5 | 18.2 | 87 | 22.1 | 18.3 | 146 |
|  | Shahi2024 (2) | 23.79 | 16.84 | 120 | 25.86 | 18.77 | 80 |
|  | Son2013 | 32 | 10.8 | 31 | 45.3 | 13.7 | 29 |
|  | Sun2014 | 20.1 | 9.4 | 38 | 21 | 8.6 | 42 |
|  | Yagi2018 | 19.8 | 11.8 | 59 | 19.2 | 12.2 | 40 |
|  | Yi2020 | 22.99 | 2.95 | 236 | 24.25 | 5.98 | 261 |
| Final follow-up | Aleksandra2014 | 22.36 | 11.18 | 50 | 16.3 | 10 | 50 |
|  | Austevoll2016 | 23.3 | 18.5 | 218 | 21 | 18.2 | 224 |
|  | Austevoll2020 | 22.2 | 18.2 | 285 | 20.5 | 17.7 | 285 |
|  | Bovonratwet2022 | 20.7 | 19.1 | 79 | 14.4 | 14.9 | 109 |
|  | Chan2018 | 21.2 | 17 | 84 | 22.6 | 20.4 | 342 |
|  | Chan2019 | 25.9 | 28 | 71 | 15.9 | 26.3 | 72 |
|  | Försth2013 | 28 | 16.6 | 4259 | 28 | 17.2 | 1131 |
|  | Försth2016 | 23.62 | 18.17 | 117 | 26.59 | 19.41 | 111 |
|  | Hua2020 | 18.8 | 2.1 | 32 | 19.4 | 2.1 | 80 |
|  | Hua2021 | 18.8 | 2 | 24 | 19.2 | 2.1 | 36 |
|  | Kim2015 | 25.68 | 14.49 | 25 | 27.2 | 12.56 | 30 |
|  | Kim2018 | 36.54 | 17.04 | 68 | 24.43 | 10.71 | 61 |
|  | Kurogochi2023 | 15.9 | 16.1 | 47 | 11.2 | 9.4 | 63 |
|  | Park2012 | 15.45 | 7.06 | 20 | 11 | 7.09 | 25 |
|  | Shahi2024 | 19.1 | 18.4 | 87 | 18.4 | 18.7 | 146 |
|  | Shahi2024 (2) | 20.19 | 18.59 | 120 | 18.91 | 16.21 | 80 |
|  | Son2013 | 25.4 | 7.9 | 31 | 25.6 | 11.5 | 29 |
|  | Sun2014 | 2.7 | 3.8 | 38 | 2.7 | 4.1 | 42 |
|  | Tozawa2022 | 14.4 | 14.2 | 18 | 17.5 | 17.4 | 24 |
|  | Yagi2018 | 18.5 | 9.5 | 59 | 14.6 | 7.8 | 40 |
|  | Yi2020 | 12.56 | 2.78 | 236 | 12.18 | 2.13 | 261 |

| Funnel plot of JOA |  |  |  |  |  |  |  |
| --- | --- | --- | --- | --- | --- | --- | --- |
|  |  | D group |  |  | F group |  |  |
|  | Author | mean | sd | total | mean | sd | total |
| Preoperative | Kurogochi2023 | 14.4 | 5.6 | 47 | 14.2 | 5.4 | 63 |
|  | Sun2014 | 14.8 | 6.7 | 38 | 15.3 | 5.8 | 42 |
|  | Tu2021 | 14.866 | 2.526 | 15 | 15.307 | 1.493 | 13 |
| Final follow-up | Kurogochi2023 | 24.5 | 4.4 | 47 | 24.4 | 3.7 | 63 |
|  | Sun2014 | 27.4 | 2.3 | 38 | 27.1 | 3 | 42 |
|  | Tu2021 | 25 | 1.414 | 15 | 22 | 2.309 | 13 |

| Funnel plot of EQ-5D |  |  |  |  |  |  |  |
| --- | --- | --- | --- | --- | --- | --- | --- |
|  |  | D group |  |  | F group |  |  |
|  | Author | mean | sd | total | mean | sd | total |
| Preoperative | Chan2018 | 0.59 | 0.22 | 84 | 0.52 | 0.23 | 342 |
|  | Chan2019 | 0.58 | 0.22 | 71 | 0.58 | 0.2 | 72 |
|  | Försth2013 | 0.36 | 0.32 | 4259 | 0.33 | 0.31 | 1131 |
|  | Försth2016 | 0.36 | 0.3 | 120 | 0.39 | 0.31 | 113 |
|  | Tozawa2022 | 0.55 | 0.13 | 18 | 0.57 | 0.17 | 24 |
| Final follow-up | Chan2018 | 0.81 | 0.16 | 84 | 0.77 | 0.2 | 342 |
|  | Chan2019 | 0.76 | 0.33 | 71 | 0.84 | 0.3 | 72 |
|  | Försth2013 | 0.62 | 0.3 | 4259 | 0.61 | 0.3 | 1131 |
|  | Försth2016 | 0.65 | 0.32 | 117 | 0.63 | 0.31 | 111 |
|  | Tozawa2022 | 0.74 | 0.15 | 18 | 0.75 | 0.18 | 24 |

| Funnel plot of complications |  |  |  |  |
| --- | --- | --- | --- | --- |
|  | D group |  | F group |  |
| Author | Number | total | Number | total |
| Austevoll2016 | 52 | 260 | 54 | 260 |
| Austevoll2020 | 42 | 285 | 45 | 285 |
| Austevoll2021 | 45 | 133 | 68 | 129 |
| Dave2019 | 22 | 37 | 10 | 27 |
| Ghogawala2016 | 2 | 35 | 1 | 31 |
| Hua2020 | 3 | 32 | 4 | 80 |
| Kleinstueck2012 | 10 | 56 | 27 | 157 |
| Kuo2019 | 24 | 164 | 68 | 437 |
| Lenga2024 | 26 | 327 | 26 | 89 |
| Son2013 | 3 | 31 | 8 | 29 |
| Staartjes2018 | 3 | 51 | 4 | 51 |
| Tu2021 | 1 | 15 | 4 | 13 |
| Ulrich2017 | 7 | 85 | 5 | 46 |
| Yi2020 | 10 | 236 | 12 | 261 |

| Funnel plot of reoperation |  |  |  |  |
| --- | --- | --- | --- | --- |
|  | D group |  | F group |  |
| Author | Number | total | Number | total |
| Austevoll2021 | 15 | 120 | 11 | 121 |
| Bovonratwet2022 | 1 | 79 | 1 | 109 |
| Chan2018 | 5 | 84 | 15 | 342 |
| Chan2019 | 10 | 71 | 1 | 72 |
| Inose2022 | 2 | 29 | 3 | 31 |
| Kuo2019 | 17 | 164 | 75 | 437 |
| Lenga2024 | 15 | 327 | 7 | 89 |
| Shafiekhani2024 | 5 | 38 | 17 | 38 |
| Shahi2024 | 7 | 87 | 11 | 146 |
| Son2013 | 2 | 31 | 3 | 29 |
| Staartjes2018 | 4 | 51 | 3 | 51 |
| Tye2016 | 32 | 227 | 24 | 137 |
| Ulrich2017 | 9 | 85 | 2 | 46 |
| Yi2020 | 17 | 236 | 15 | 261 |

| Funnel plot of Odom’s criteria |  |  |  |  |
| --- | --- | --- | --- | --- |
|  | D group |  | F group |  |
| Author | Number | total | Number | total |
| Lin2019 | 22 | 33 | 15 | 16 |
| Park2012 | 13 | 20 | 14 | 25 |
| Son2013 | 26 | 31 | 23 | 29 |

2024/08/15 (YXC, WL)
